# Turn to TCRs when CARs fail

**DOI:** 10.18632/oncotarget.15088

**Published:** 2017-02-04

**Authors:** Hans J. Stauss

**Affiliations:** Institute of Immunity and Transplantation, Royal Free Campus, University College London, London

**Keywords:** immunotherapy, CD20, T cell receptor, gene therapy, T cells, B cell tumors

The past few years have brought exciting progress in the development of novel forms of immunotherapy of cancer. While antibody blockade of negative pathways that inhibit T cell function, such as CTLA-4 and PD1, has resulted in clinical benefits for patients who have failed conventional therapy options, this strategy lacks cancer specificity as it also activates T cells with self-reactive potential. Hence, autoimmunity is a serious side effect of anti-CTLA4 and anti-PD1 pathway therapy [1]. Engineering T cell specificity provides a strategy to direct immunity towards defined cancer-specific or cancer-associated antigens, thus improving tumor immunity and decreasing the risk of autoimmunity.

Engineering specificity is most commonly achieved with retroviral and lentiviral gene transfer vectors that equip patient T cells with therapeutic T cell receptors (TCRs) or with chimeric antigen receptors (CARs). CARs contain the antigen-binding domains of antibody molecules fused to transmembrane and cytosolic domains required for surface expression and for triggering T cell function. The major advantage of CARs is the direct recognition of cancer-antigens irrespective of the HLA genotype of patients. Hence, the most successful CARs that target CD19, a marker of lymphocytes of the B-cell linage, provides a treatment option for all patients with CD19-positive B-cell malignancies. In contrast, TCRs recognize peptide fragments that are presented by highly polymorphic HLA molecules. Successful TCR based therapy therefore requires not only the expression of the relevant antigen in cancer cells but also the selection of patients with appropriate HLA alleles. However, a distinct advantage of TCRs is that they mediate T cell activation when triggered by low concentration of antigen, while T cell stimulation by CARs requires relatively high antigen expression in cancer cells.

Jahn and colleagues have tested whether TCRs can indeed mediate T cell activation by low antigen concentration that would be expected to be insufficient for CAR-mediated T cell activation [6]. They have raised TCRs against CD20, a B-linage marker that has been successfully targeted by therapeutic antibodies. In order to circumvent tolerance to CD20, the investigators took advantage of the allo-restricted strategy to isolate CD20-specific T cells from the non-tolerant TCR repertoire of allogeneic healthy donors [2]. Using this strategy they have raised CD20-specific T cells restricted by HLA-A*02:01 and B*07:02, two HLA alleles that are frequent in the Caucasian population. They went on to demonstrate that the CD20-specific T cells were able to recognize and efficiently kill B-linage cancer cells expressing low levels of the target antigen. In contrast, CD20-specific antibodies were unable to mediate complement killing of B-cell malignancies expressing low levels of antigen; successful antibody attack was only seen against cancer cells with high CD20 expression. Following cloning of the genes encoding the TCR alpha and beta chains of the CD20-specific T cells, the authors showed that retroviral TCR gene transfer readily converted peripheral lymphocytes into therapeutic T cells that effectively killed B-linage cancer cells irrespective of the CD20 expression levels.

This study suggests that T cell engineering with TCRs and CARs can be complementary. Although Jahn and colleagues have not directly compared TCRs and CARs with specificity for the same target antigen, previously published work has shown that as few at one antigenic peptide epitope can be sufficient for TCR mediated T cell activation [3, 4]. In contrast, more than 1000 copies of antigen are usually required when CARs mediate T cell activation [5]. Therefore, the down-modulation of antigen expression levels in patients treated with CAR therapy may enable the malignant cells to escape attack by CAR-expressing T cells. However, such antigen low escape variants would be expected to remain susceptible to TCR attack, suggesting that TCR gene therapy may be an effective treatment option for patients with tumors that have escaped destruction by CAR engineered T cells. When combined with allogeneic stem cell transplantation, which is often used to treat hematological malignancies, TCR gene therapy can offer a strategy to selectively attack malignant cells but not normal B cells. This can be achieved by selecting stem cells, reconstituting the normal B-cell compartment, from donors who do not express the HLA allele required for TCR recognition. Consequently, the HLA-restricted TCR recognition directs T cell attack against patient malignant B cells and spares donor-derived B cells, providing a level of specificity that cannot be achieved with CARs.
